# Immune Checkpoints in Circulating and Tumor-Infiltrating CD4^+^ T Cell Subsets in Colorectal Cancer Patients

**DOI:** 10.3389/fimmu.2019.02936

**Published:** 2019-12-17

**Authors:** Salman M. Toor, Khaled Murshed, Mahmood Al-Dhaheri, Mahwish Khawar, Mohamed Abu Nada, Eyad Elkord

**Affiliations:** ^1^Cancer Research Center, Qatar Biomedical Research Institute (QBRI), Hamad Bin Khalifa University (HBKU), Qatar Foundation (QF), Doha, Qatar; ^2^Department of Pathology, Hamad Medical Corporation, Doha, Qatar; ^3^Department of Surgery, Hamad Medical Corporation, Doha, Qatar; ^4^Biomedical Research Centre, School of Science, Engineering and Environment, University of Salford, Salford, United Kingdom

**Keywords:** colorectal cancer, T regulatory cells, immune checkpoints, tumor microenvironment, T cells

## Abstract

Blockade of inhibitory immune checkpoints (ICs) is a promising therapeutic approach; however, it has shown limited success in some cancers including colorectal cancer (CRC). The tumor microenvironment (TME) is largely responsible for response to therapy, and its constituents may provide robust biomarkers for successful immunotherapeutic approaches. In this study, we performed phenotypical characterization and critical analyses of key inhibitory ICs and T regulatory cell (Treg)-related markers on CD4^+^ T cell subsets in CRC patients, and compared with normal colon tissues and peripheral blood from the same patients. We also investigated correlations between the levels of different CD4^+^ T cell subsets and the clinicopathologic features including disease stage and tumor budding. We found a significant increase in the levels of CD4^+^FoxP3^+^Helios^+^ T cells, which represent potentially highly immunosuppressive Tregs, in the CRC TME. Additionally, tumor-infiltrating CD4^+^ T cells upregulated programmed cell death protein-1 (PD-1), cytotoxic T-lymphocyte-associated protein-4 (CTLA-4), T cell immunoglobulin and mucin domain-3 (TIM-3) and lymphocyte-activation gene 3 (LAG-3). We also characterized the expression of PD-1, CTLA-4, TIM-3, and LAG-3 on different CD4^+^FoxP3^−/+^Helios^−/+^ T cell subsets. Interestingly, we found that CTLA-4, TIM-3, and LAG-3 were mainly co-expressed on FoxP3^+^Helios^+^ Tregs in the TME. Additionally, FoxP3^high^ Tregs expressed higher levels of Helios, CTLA-4 and TIM-3 than FoxP3^low^ T cells. These results highlight the significance of Tregs in the CRC TME and suggest that Tregs may hamper response to IC blockade in CRC patients, but effects of different IC inhibition regimes on Treg levels or activity warrants further investigations. We also found that CD4^+^CTLA-4^+^ T cells in circulation are increased in patients with advanced disease stage. This study simultaneously provides important insights into the differential levels of CD4^+^ T cell subpopulations and IC expression in CRC TME, compared to periphery and associations with clinicopathologic features, which could be used as potential biomarkers for CRC progression and response to therapy.

## Introduction

The tumor immune microenvironment (TIME) is largely accountable for response to immunotherapeutic modalities, and better analyses of its constituents can help develop robust biomarkers to identify patients who would respond to immunotherapy (1). In addition, DNA fragments or tumor cells budded off from the primary tumor sites may also be detected in “liquid biopsies” and used as potential biomarkers for initiation of effective anti-tumor therapies (2, 3).

Colorectal cancer (CRC) is among the leading causes of cancer-related mortality and morbidity worldwide, affecting ~1.4 million newly diagnosed patients and causing death in 0.7 million every year (4, 5). Current treatments for primary and metastatic CRC primarily include laparoscopic surgeries, radiotherapy, and neoadjuvant and palliative chemotherapies (6, 7). Immunotherapy regimes however have not had a big impact on treating CRC as in treating other malignancies. Nonetheless, pembrolizumab, an immune checkpoint (IC) inhibitor [anti-programmed cell death protein 1 (PD-1)], was recently granted Food and Drug Authority (FDA) approval for treating unresectable or metastatic solid tumors, including CRC, with microsatellite instability high (MSI-H) or DNA mismatch repair deficiency (dMMR) (8, 9).

Inhibitory ICs attenuate T cell responses to mediate immune tolerance (10). These immune-inhibitory pathways are often employed by tumors to facilitate immune evasion. High Treg infiltration coupled with high IC expression in the tumor microenvironment (TME) should further promote tumor progression due to T cell exhaustion and impaired cytokine release. Several studies have reported accumulation of highly suppressive Treg populations and elevated IC expression in the colorectal TME (11–13). However, accumulation of Tregs in CRC patients can have opposing effects on prognosis also as it may be associated with favorable clinical outcomes (14, 15).

In this study, we investigated the immune landscape of colorectal tumors, compared to normal colon tissues and peripheral blood from the same patients. We focused our investigations on CD4^+^ T cells and on the expression of key inhibitory ICs and regulatory T cell (Treg)-related markers. Tumor-specific T cells are a key component of the TME due to the presence of a multitude of suppressive mechanisms within the TME, which assist tumor immune evasion. Accumulation of Tregs within the TME leads to an immune-permissive microenvironment, favoring uncontrolled tumor growth (16, 17). Potent anti-tumor immune responses require a shift in balance between levels of Tregs and T effector cells (Teff) in the TME (18). Therefore, T cell trafficking and localization into tumor sites and preferential proliferation and differentiation of tumor-reactive T cells can facilitate effective immunotherapies (19). Moreover, T cell inflamed tumors, characterized by existing anti-tumor T cell responses, are associated with improved clinical outcomes in CRC patients (11, 20).

We found a significant increase in CD4^+^ T cells in the CRC TME, compared with adjacent normal tissue. Moreover, these CD4^+^ T cells comprised of potentially suppressive FoxP3^high^ Treg populations, which co-expressed high levels of Helios, previously reported as a marker for activated Tregs (21). Additionally, we found that intratumoral CD4^+^ T cells upregulate multiple inhibitory ICs including PD-1, cytotoxic T-lymphocyte-associated protein-4 (CTLA-4), T cell immunoglobulin and mucin domain-3 (TIM-3), and lymphocyte-activation gene 3 (LAG-3). We also compared the levels of different CD4^+^ T cell subsets between CRC patients presenting with early and advanced stage disease, and between patients who showed varying tumor budding status. We found that patients with advanced stage disease have increased CTLA-4 expression on CD4^+^ T cells in circulation. Overall, this study increases our knowledge about the potential use of checkpoint blockade in CRC patients.

## Materials and Methods

### Sample Collection and Storage

This study was performed under ethical approvals from Qatar Biomedical Research Institute, Doha, Qatar (Protocol no. 2018-018) and Hamad Medical Corporation, Doha, Qatar (Protocol no. MRC-02-18-012). All experiments were performed in accordance with relevant guidelines and regulations.

Peripheral blood samples were collected in EDTA tubes from 34 CRC patients, and tumor tissues (TT) and paired, adjacent non-cancerous normal colon tissues (NT) were obtained from 27 out of these 34 patients, who underwent surgery at Hamad Medical Corporation, Doha, Qatar. All patients included in the study were treatment-naïve prior to surgery and provided written informed consent prior to sample collection. Table 1 shows the clinical and pathological characteristics of the study population.

**Table 1:** Characteristic features of study populations.

	**CRC patients**
Number	34 (27)^†^
Age (median)	62 (31-96)^§^
Gender (Male: Female)	24:10
**TNM stage**	
I	6 (1)^†^
II	10 (10)^†^
III	15 (13)^†^
IV	3 (3)^†^
DNA mismatch repair deficiency (dMMR)	4 (3)^†^
**Tumor budding**	
Low	13 (11)^†^
Intermediate	11 (7)^†^
High	10 (9)^†^
**Histological grade**	
G2 Moderately differentiated	All samples

*CRC; Colorectal cancer*.

§ *Median range*.

† *Samples used for analyses of tumor-infiltrating immune cells*.

Peripheral blood mononuclear cells (PBMC) were isolated from fresh blood by density-gradient centrifugation using Histopaque-1077 (Sigma-Aldrich, St. Louis, MO, USA). PBMC were frozen in freezing media (50% FBS, 40% RPMI 1640 media and 10% DMSO) at a density of 5 million cells per 1 ml in cryovials to be used in batches for subsequent analyses. Tissue specimens were also stored in freezing media for subsequent analyses.

### Cell Isolation From Colorectal Tumors and Normal Colon Tissues

Cells were isolated from NT and TT by mechanical disaggregation. Briefly, tissues frozen in freezing media were thawed and washed with phosphate-buffered saline (PBS) and then mechanically cut into small pieces (~2–4 mm) using a surgical scalpel. Tissue disaggregation was performed on a gentleMACS dissociator (Miltenyi Biotech, Bergisch Gladbach, Germany) without using enzymes. The cell suspension was then passed through a 100 μM cell strainer to remove aggregates and debris. The single cell suspension was washed with PBS and stained for flow cytometric analyses.

### Multi-Parametric Flow Cytometry

PBMC and cells isolated from tissues were washed with PBS and re-suspended in 100 μl flow cytometry staining buffer (PBS with 1% FCS and 0.1% sodium azide). Fc receptors (FcR) were first blocked using FcR Blocker (Miltenyi Biotec). Fixable Viability Dye eFluor 780 (eBioscience, San Diego, USA) was added to gate live cells. Cells were then stained with cell surface antibodies including CD3-Alexa Fluor 700 (clone UCHT-1; BD Biosciences, Oxford, UK), CD4-phycoerythrin (clone RPA-T4; BD Biosciences), CD25-Brilliant Violet 650 (clone BC96; BioLegend, San Diego, USA), PD-1-PE/Dazzle™ 594 (clone EH12.2H7; BioLegend), LAG-3-Brilliant violet 421 (clone T47-530; BD Biosciences) and TIM-3-Brilliant Violet 711 (clone 7D3; BD Biosciences) and incubated at 4°C for 30 min. Cells were then washed twice with flow cytometry staining buffer. For intracellular staining, cells were incubated at 4°C for 45 min in fixation/permeabilization buffer (eBioscience). Cells were then washed twice with permeabilization wash buffer (eBioscience). Mouse serum (Sigma-Aldrich) and rat serum (Sigma-Aldrich) were added to block non-specific binding sites for 10 min at 4°C. Intracellular antibodies including CTLA-4-PerCP-eFluor 710 (clone 14D3; eBioscience), FoxP3-phycoerythrin cyanin 7 (PE/Cy7) (clone PCH101; eBioscience) and Helios-Fluorescein Isothiocyanate (FITC) (clone 22F6; BioLegend) were then added and cells incubated for another 30 min at 4°C. Cells were then washed twice with permeabilization wash buffer (eBioscience), and re-suspended in flow cytometry staining buffer.

All data were acquired on a BD LSRFortessa X-20 SORP flow cytometer using BD FACSDiva software (BD Biosciences) and analyzed on FlowJo V10 software (FlowJo, Ashland, USA).

### Statistical Analyses

Statistical analyses were performed using GraphPad Prism 8 software (GraphPad Software, California, USA). One-way Anova test was performed to check for statistical significance in grouped analyses. Paired *t*-tests were performed within groups on samples that passed the Shapiro-Wilk normality test, while Wilcoxon matched-pairs signed rank tests were performed on samples that did not show normal distribution. Unpaired *t*-tests were performed for comparisons between groups on normally distributed data and Mann-Whitney tests for samples that did not show normal distribution. A *P* > 0.05 was considered statistically non-significant. The *P*-values are represented as follows; ^***^P < 0.001, ^**^P < 0.01, ^*^P < 0.05. Data are presented as mean ± standard error of the mean (SEM).

## Results

### Increased Levels of CD4^+^ T Cells in the Tumor Microenvironment of Colorectal Cancer Patients

**Figure 1:**
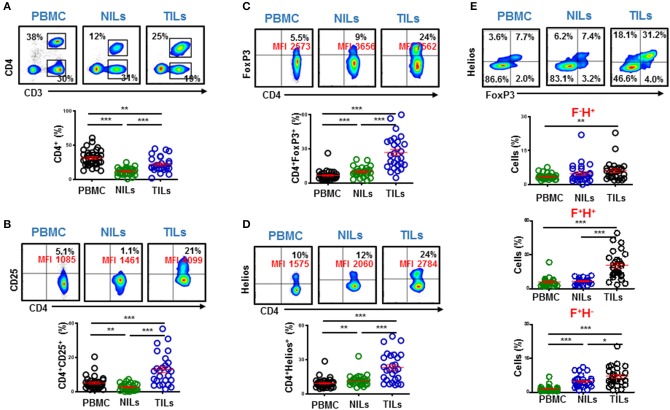
Levels of circulating and tumor-infiltrating CD4^+^ T cells in colorectal cancer patients and expression levels of T regulatory cell-related markers. PBMC from 34 colorectal cancer (CRC) patients and immune cells isolated from colorectal tumor tissues (TILs) and paired, adjacent non-tumor normal colon tissues (NILs) from 27 patients were stained for CD4^+^ T cell markers and Treg-related markers including CD25, FoxP3, and Helios. Representative flow cytometric plots and scatter plots show the levels of CD3^+^CD4^+^ T cells in PBMC, NILs, and TILs from CRC patients **(A)**. Representative flow cytometric and scatter plots show expression levels of CD25 **(B)**, FoxP3 **(C)**, and Helios **(D)** in CD4^+^ T cells in PBMC, NILs, and TILs. Representative flow cytometric plots show FoxP3 and Helios co-expression and scatter plots show differences in levels of CD4^+^FoxP3^−/+^Helios^−/+^ subsets in PBMC, NILs and TILs **(E)**. ^***^P < 0.001, ^**^P < 0.01, ^*^P < 0.05.

Accumulation of tumor-infiltrating T cells in CRC patients has been previously reported, and shown to be associated with favored clinical outcomes (11). We investigated the levels of CD4^+^ T cells in circulation, normal colon tissues and in the TME of CRC patients. The overall levels of circulating CD4^+^ and CD4^−^ T cells in our cohort were similar (CD4^+^; 31.4% vs. CD4^−^; 31.7%, Figure 1A). In agreement with previous reports, we found that CD4^+^ T cells accumulate in colorectal tumors, compared with normal tissues but were lower compared to their levels in circulation (PBMC; 31.4 ± 2.0 vs. NILs; 11.5 ± 1.0 vs. TILs; 22.0 ± 2.1, Figure 1A). Levels of Teff cells within the TME can greatly affect cancer progression and response to therapy (18). Therefore, we focused our subsequent investigations to perform further phenotypical characterization of CD4^+^ T cell subsets to ascertain their role in colorectal tumor biology.

### Tumor-Infiltrating CD4^+^ T Cells in CRC Patients Comprise Mainly of Potentially Suppressive T Regulatory Cells

Tregs constitute an important subset of CD4^+^ T cells, which are characterized by high expression of interleukin-2 receptor alpha chain (CD25) and forkhead box P3 (FoxP3) transcription factor (22). Moreover, Helios is a key transcription factor, which regulates FoxP3^+^ Treg functional stability and it is required for their inhibitory activity (23). Infiltration of FoxP3^+^ Tregs is often associated with poor prognosis and disease progression (24). We found that the levels of CD4^+^CD25^+^, CD4^+^FoxP3^+^ and CD4^+^Helios^+^ T cells were significantly higher in the TME, compared with NT and circulation (CD25: PBMC; 5.0 ± 0.6 vs. NILs; 2.6 ± 0.4 vs. TILs; 13.0 ± 1.8, FoxP3: 6.5 ± 0.7 vs. 9.8 ± 0.8 vs. 26.8 ± 2.8 & Helios: 9.1 ± 0.8 vs. 11.8 ± 1.0 vs. 23.1 ± 2.5, Figures 1B–D). We also found that Tregs in CRC TME comprise mainly of FoxP3^+^Helios^+^ Tregs, which were significantly higher in the TME compared with normal tissue and periphery (5.7 ± 0.6 vs. 6.6 ± 0.5 vs. 20.5 ± 2.3, Figure 1E).

**Figure 2:**
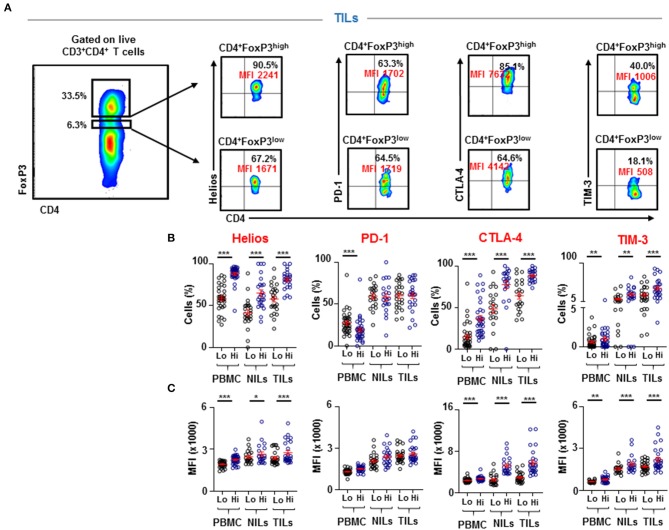
Differences in Helios and immune checkpoint expression on CD4^+^FoxP3^low^ and CD4^+^FoxP3^high^ PBMC, NILs, and TILs from CRC patients. CD4^+^FoxP3^low^ and CD4^+^FoxP3^high^ Tregs were gated in PBMC, NILs, and TILs to investigate Helios, PD-1, CTLA-4, and TIM-3 expressions. Representative flow cytometric plots show gating strategy for defining CD4^+^FoxP3^low/high^ Tregs and the expression levels of Helios, PD-1, CTLA-4, and TIM-3, in TILs isolated from CRC patients **(A)**. Scatter plots show differences in expression level **(B)** and Mean Fluorescence Intensity (MFI) **(C)** of Helios, PD-1, CTLA-4, and TIM-3 between CD4^+^FoxP3^low^ and CD4^+^FoxP3^high^ PBMC, NILs, and TILs from CRC patients. ^***^P < 0.001, ^**^P < 0.01.

FoxP3^high^ Tregs have been previously identified as suppression-competent, while FoxP3^low^ T cells identified as non-suppressive Tregs (14). Therefore, we investigated Helios expression within FoxP3^high^ and FoxP3^low^ populations to ascertain the potential suppressive characteristics of the FoxP3^+^Helios^+^ subpopulation, accumulated in CRC tumors. We found that CD4^+^FoxP3^high^ Tregs express significantly higher levels of Helios than CD4^+^FoxP3^low^ cells in PBMC, NILs, and TILs (PBMC; 58.8 ± 2.6 vs. 88.1 ± 1.7, NILs; 41.4 ± 3.7 vs. 65.1 ± 3.9 & TILs; 58.0 ± 3.5 vs. 80.9 ± 2.3, Figure 2). Next, we investigated differences in expression levels of different inhibitory ICs between FoxP3^low^ and FoxP3^high^ Tregs. Interestingly, we found that CTLA-4 and TIM-3 were also expressed at significantly higher levels on CD4^+^FoxP3^high^ Tregs than CD4^+^FoxP3^low^ T cells in PBMC, NILs, and TILs (CTLA-4: PBMC; 15.1 ± 2.7 vs. 36.7 ± 3.6, NILs; 48.5 ± 5.2 vs. 77.6 ± 4.8, TILs; 64.4 ± 4.7 vs. 88.3 ± 1.5 & TIM-3: PBMC; 0.6 ± 0.1 vs. 1.0 ± 0.3, NILs; 11.3 ± 2.5 vs. 22.7 ± 3.6, TILs; 21.6 ± 3.7 vs. 38.2 ± 4.7, Figure 2). However, PD-1 did not show any significant differences in expression levels between CD4^+^FoxP3^low/high^ NILs and TILs, but it was significantly lower on CD4^+^FoxP3^high^ T cells than CD4^+^FoxP3^low^ T cells in circulation (PBMC; 27.5 ± 2.5 vs. 19.1 ± 2.4, Figure 2).

We also compared the mean fluorescence intensity (MFI) of the different markers between CD4^+^FoxP3^low^ and CD4^+^FoxP3^high^ Tregs (Figure 2C). We found that the differences in population frequencies were also reflected in differences in MFI. The MFI for Helios was significantly higher in CD4^+^FoxP3^high^ Tregs than CD4^+^FoxP3^low^ T cells in PBMC (1928 ± 36.6 vs. 2253 ± 52.1), NILs (2457 ± 108.2 vs. 2656 ± 163.7) and TILs (2374 ± 97.4 vs. 2759 ± 156.9). CTLA-4 and TIM-3 also showed similar patterns (CTLA-4: PBMC; 2384 ± 64.5 vs. 2701 ± 73.2, NILs; 2492 ± 221.1 vs. 5197 ± 365.8, TILs; 2922 ± 188.5 vs. 5797 ± 501.5 and TIM-3: PBMC; 621 ± 19.5 vs. 771 ± 44.5, NILs; 1546 ± 84.9 vs. 1889 ± 145.0, TILs; 1708 ± 69.6 vs. 2203 ± 161.2), while PD-1 did not show any significant differences in MFI between CD4^+^FoxP3^low^ and CD4^+^FoxP3^high^ Tregs in PBMC, NILs and TILs (Figure 2C).

### High Expression of Immune Checkpoints on Intratumoral CD4^+^ T Cells

**Figure 3:**
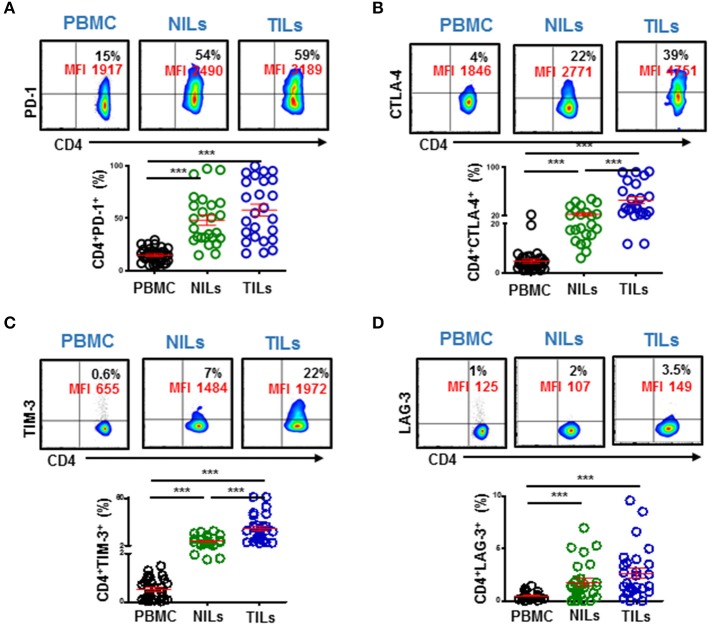
Immune checkpoint expression on CD4^+^ T cells in PBMC, NILs, and TILs from CRC patients. PBMC, NILs, and TILs were stained for CD3, CD4, and key immune checkpoints. Representative flow cytometric plots and scatter plots show differences in expression levels of PD-1 **(A)**, CTLA-4 **(B)**, TIM-3 **(C)**, and LAG-3 **(D)** on CD4^+^ T cells in PBMC, NILs, and TILs. ^***^P < 0.001.

Immune checkpoints are expressed on activated or exhausted T cells (16). To find out the functional state of infiltrating T cells in the colorectal TME, we investigated IC expression on different CD4^+^ T cell subsets. We found that key inhibitory ICs, including PD-1, CTLA-4, TIM-3 and LAG-3 were highly expressed on CD4^+^ TILs (Figure 3). These ICs were expressed at significantly lower levels in periphery compared to normal colon tissue and showed elevated expression levels in the TME (PD-1: PBMC; 14.9 ± 1.1 vs. NILs; 48.1 ± 4.8 vs. TILs; 57.8 ± 5.7, CTLA-4: 4.8 ± 0.8 vs. 22.7 ± 2.4 vs. 45.9 ± 5.7, TIM-3: 0.5 ± 0.1 vs. 7.5 ± 1.0 vs. 23.2 ± 3.2 & LAG-3: 0.4 ± 0.1 vs. 1.8 ± 0.4 vs. 2.7 ± 0.5, Figure 3). Moreover, it is noteworthy that the overall expression levels of PD-1 were highest in tissues and in periphery, followed by CTLA-4 and TIM-3, while LAG-3 showed lowest overall expression on CD4^+^ T cells compared to other ICs (Figure 3).

**Figure 4:**
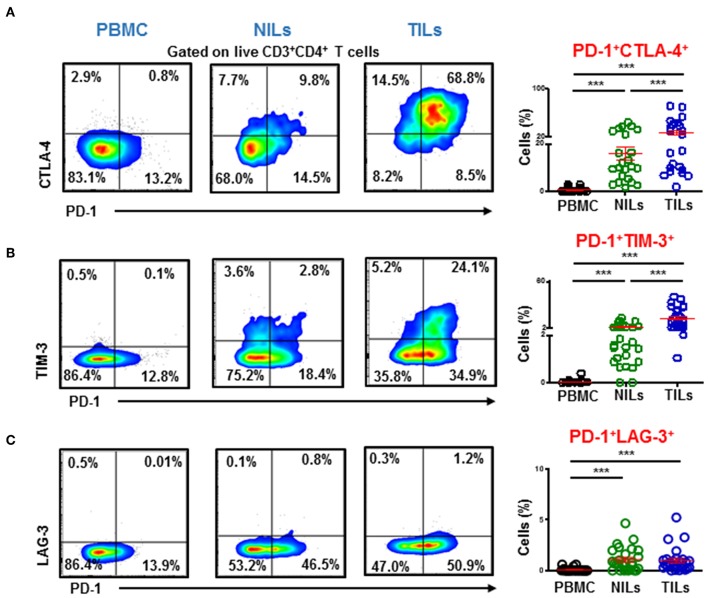
Immune checkpoints co-expression on CD4^+^ T cells. PBMC, NILs, and TILs isolated from CRC patients were stained for CD3, CD4, and immune checkpoints. Representative flow cytometric and scatter plots show differences in co-expression levels of PD-1^−/+^CTLA-4^−/+^ **(A)**, PD-1^−/+^TIM-3^−/+^ **(B)**, and PD-1^−/+^LAG-3^−/+^ **(C)** on CD4^+^ T cells in PBMC, NILs, and TILs. ^***^P < 0.001.

We also investigated co-expression of PD-1 with other ICs in PBMC, NILs, and TILs. We found that PD-1 was mainly co-expressed with CTLA-4 and TIM-3 in CD4^+^ TILs (Figures 4A,B). In contrast, although CD4^+^PD-1^+^LAG-3^+^ T cells were significantly higher in NILs and in TILs compared to PBMC, the majority of CD4^+^PD-1^+^ T cells do not co-express LAG-3 (Figure 4C).

### CTLA-4, TIM-3, and LAG-3 Are Mainly Expressed on FoxP3^+^Helios^+^ Tregs in the Tumor Microenvironment

**Figure 5:**
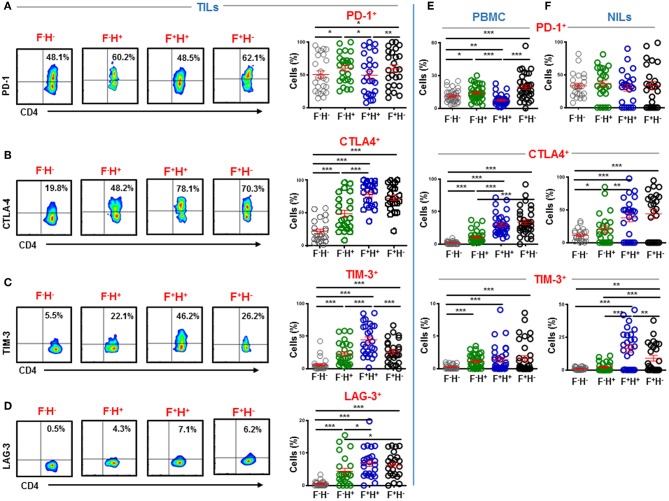
Immune checkpoint expression on FoxP3 and Helios subsets in CD4^+^ TILs, PBMC, and NILs. TILs isolated from CRC patients were stained for CD3, CD4, immune checkpoints and FoxP3 and Helios. Representative flow cytometric plots and scatter plots show differences in expression levels of PD-1 **(A)**, CTLA-4 **(B)**, TIM-3 **(C)**, and LAG-3 **(D)** in CD4^+^FoxP3^−/+^Helios^−/+^ TILs subsets. Scatter plots show differences in expression levels of PD-1, CTLA-4, and TIM-3 in CD4^+^FoxP3^−/+^Helios^−/+^ PBMC **(E)** and NILs **(F)** from CRC patients. ^***^P < 0.001, ^**^P < 0.01, ^*^P < 0.05.

A plausible approach to evoke potent antitumor immune responses without triggering autoimmunity is to target terminally differentiated Tregs (24). Previous studies have reported overexpression of various ICs on Tregs including constitutive expression of CTLA-4 (25–27). We wanted to identify which Treg subpopulations upregulate inhibitory ICs in the colorectal TME based on FoxP3 and Helios expression. We found that key inhibitory ICs are differentially upregulated on various intratumoral Treg subsets, compared to CD4^+^FoxP3^−^Helios^−^ non-Tregs (Figures 5A–D). In TILs, PD-1 was mainly expressed on CD4^+^FoxP3^−^Helios^+^ (60.3 ± 4.5) and CD4^+^FoxP3^+^Helios^−^ Tregs (59.7 ± 5.5), compared to CD4^+^FoxP3^+^Helios^+^ (49.5 ± 6.1) and CD4^+^FoxP3^−^Helios^−^ (50.6 ± 5.5) (Figure 5A). CTLA-4 and LAG-3 were mainly expressed on both CD4^+^FoxP3^+^Helios^+^ (CTLA-4; 78.5 ± 3.8 & LAG-3; 6.8 ± 0.9) and CD4^+^FoxP3^+^Helios^−^ Tregs (CTLA-4; 72.8 ± 4.1 & LAG-3; 6.2 ± 0.8), compared to CD4^+^FoxP3^−^Helios^+^ TILs (CTLA-4; 48.7 ± 5.6 & LAG-3; 4.4 ± 0.9) and CD4^+^FoxP3^−^Helios^−^ TILs (CTLA-4; 21.8 ± 3.5 & LAG-3; 0.5 ± 0.1) (Figures 5B,D). While, TIM-3 was mainly expressed on CD4^+^FoxP3^+^Helios^+^ TILs (44.5 ± 4.6), compared to CD4^+^FoxP3^−^Helios^+^ (22.8 ± 3.4), CD4^+^FoxP3^+^Helios^−^ TILs (25.9 ± 3.4) and CD4^+^FoxP3^−^Helios^−^ TILs (5.9 ± 1.8) (Figure 5C).

Notably, inhibitory ICs showed different expression patterns on CD4^+^FoxP3^−/+^Helios^−/+^ in periphery. PD-1 was mainly expressed on CD4^+^FoxP3^+^Helios^−^ Tregs in circulation (19.5 ± 2.0), followed by CD4^+^FoxP3^−^Helios^+^ T cells (14.0 ± 1.1) and CD4^+^FoxP3^−^Helios^−^ T cells (11.3 ± 1.0), while CD4^+^FoxP3^+^Helios^+^ Tregs showed lowest PD-1 expression (7.4 ± 0.8) (Figure 5E). CTLA-4 was mainly expressed on CD4^+^FoxP3^+^Helios^−^ (33.8 ± 3.5) and CD4^+^FoxP3^+^Helios^+^ Tregs (30.0 ± 2.7), compared to CD4^+^FoxP3^−^Helios^+^ (10.7 ± 1.6) and CD4^+^FoxP3^−^Helios^−^ T cells (1.9 ± 0.3). TIM-3 was mainly expressed on FoxP3 and/or Helios-expressing T cells, compared to CD4^+^FoxP3^−^Helios^−^ T cells (0.2 ± 0.1) in circulation (Figure 5E). In addition, no significant differences were detected in TIM-3 expression on CD4^+^FoxP3^−^Helios^+^ (1.1 ± 0.2), CD4^+^FoxP3^+^Helios^+^ (1.4 ± 0.4) and CD4^+^FoxP3^+^Helios^−^ (1.6 ± 0.4) T cells in circulation (Figure 5E). In normal colon tissues, PD-1 expression did not show any significant differences in CD4^+^FoxP3^−^Helios^+^ (36.4 ± 5.0), CD4^+^FoxP3^+^Helios^+^ (30.2 ± 5.0), CD4^+^FoxP3^+^Helios^−^ (33.8 ± 6.4) and CD4^+^FoxP3^−^Helios^−^ (33.3 ± 3.8) NILs (Figure 5F). CTLA-4 was also mainly expressed on CD4^+^FoxP3^+^Helios^−^ (43.9 ± 7.0) and CD4^+^FoxP3^+^Helios^+^ NILs (37.9 ± 6.1), compared to CD4^+^FoxP3^−^Helios^+^ (20.5 ± 4.8) and CD4^+^FoxP3^−^Helios^−^ NILs (11.1 ± 1.9). In contrast, TIM-3 was mainly expressed on CD4^+^FoxP3^+^Helios^+^ NILs (16.6 ± 2.9), followed by CD4^+^FoxP3^+^Helios^−^ (8.7 ± 2.1) and CD4^+^FoxP3^−^Helios^+^ (2.1 ± 0.7) NILs, while CD4^+^FoxP3^−^Helios^−^ NILs showed minimal TIM-3 expression (1.0 ± 0.2) (Figure 5F). Of note, there were no conclusive results to identify the CD4^+^FoxP3^+/−^Helios^+/−^ subset with highest LAG-3 expression due to weak overall LAG-3 expression in periphery (data not shown).

**Figure 6:**
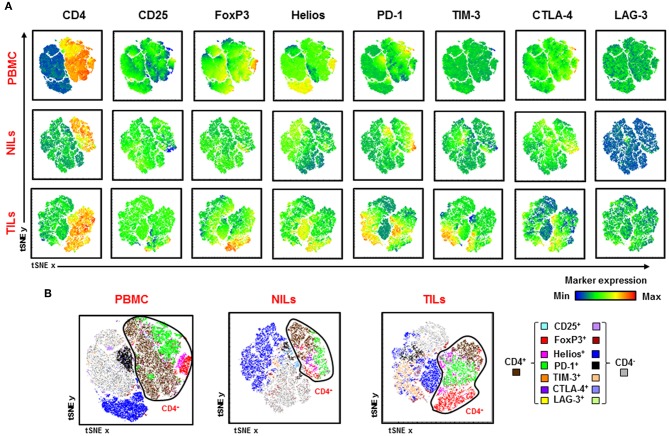
Visualizing expression of Treg-related markers and immune checkpoint expression in PBMC, NILs, and TILs from CRC patients. Flow cytometric data from PBMC, NILs, and TILs were merged to create single t-Distributed Stochastic Neighbor Embedding (tSNE) maps. Cells (dots) are plotted according to expression of CD4, CD25, FoxP3, Helios, PD-1, TIM-3, CTLA-4, and LAG-3 in PBMC, NILs, and TILs from CRC patients **(A)**. tSNE maps show combined co-expression of the different Treg-related markers and immune checkpoints on CD4^+^ and CD4^−^ T cells in PBMC, NILs and TILs from CRC patients **(B)**.

Visualization of overall CD4^+^ T cell infiltrates in CRC tumors and periphery is depicted in Figure 6. We generated t-Distributed Stochastic Neighbor Embedding (tSNE) plots from various Treg-related markers and inhibitory ICs in PBMC, NILs, and TILs from CRC patients. We confirmed that FoxP3, Helios, PD-1, TIM-3, and CTLA-4 expression levels are increased in CD4^+^ TILs, while CD4^+^ T cells showed lower Treg-related markers and IC expression in PBMC and NILs (Figure 6A). In addition, CD4^+^ TILs co-expressed multiple ICs compared to PBMC and NILs (Figure 6B). Helios, PD-1 and TIM-3 were also expressed on CD4^−^ T cells and showed elevated expression on CD4^−^ TILs (Figure 6B).

### Patients With Advanced Stage Disease Have Higher Levels of CD4^+^CTLA-4^+^ T Cells in Circulation

**Figure 7:**
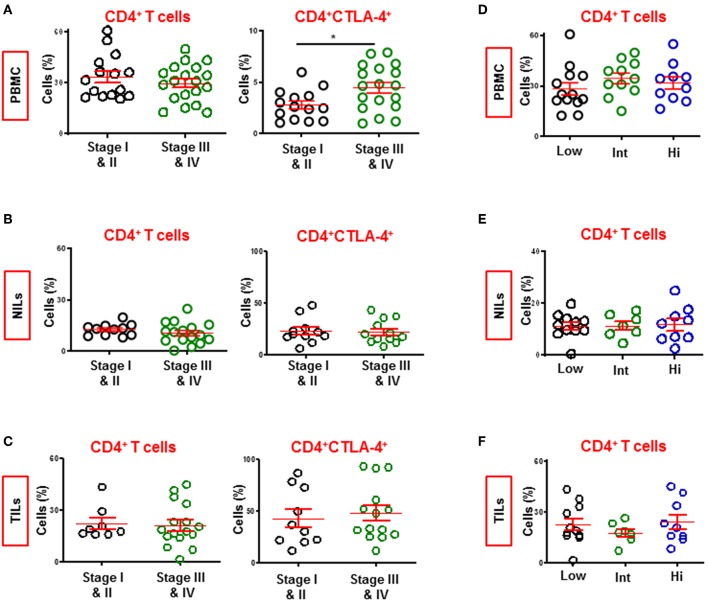
Levels of CD4^+^ and CD4^+^CTLA-4^+^ T cells in CRC patients with different staging and tumor budding. Patients were divided into two groups based on pathologic staging; early stage (Stage I and II) and advanced stage (Stage III and IV). Scatter plots show differences in levels of CD4^+^ and CD4^+^CTLA-4^+^ T cells in PBMC **(A)**, NILs **(B)**, and TILs **(C)** between CRC patients with early and advanced stages. Patients were also divided into three groups based on degree of tumor budding; low, intermediate and high. Scatter plots show differences in levels of CD4^+^ T cells in PBMC **(D)**, NILs **(E)**, and TILs **(F)** between CRC patients. ^*^P < 0.05.

We compared the levels of CD4^+^ T cell subsets in circulation, NILs and TILs between CRC patients presenting with early and advanced pathologic stages (Figures 7A–C). We combined patients with stages I and II (PBMC; *n* = 15, NILs/TILs; *n* = 11) and compared with those with stages III and IV advanced stage (PBMC; *n* = 19, NILs/TILs; *n* = 16). We found that the levels of circulating CD4^+^ T cells were similar between patients with early or advanced stages. Intestinally, there was a significant increase in levels of CD4^+^CTL-4^+^ T cells only in circulation of patients with advanced stage (4.0 ± 1.3 vs. 5.4 ± 1.1, Figure 7A). The constitutive expression of CTLA-4 on CD4^+^ Tregs suggests correlation of elevated levels of CTLA-4^+^ Tregs with CRC disease progression. However, other T cell subsets, including CD4^+^FoxP3^+^ Tregs, did not show any correlation with disease progression (data not shown).

### Levels of CD4^+^ T Cells Are Similar in Patients Exhibiting Varying Tumor Budding Status

Tumor budding in CRC has been associated with poor disease outcomes in various studies (28). We divided our cohort into three groups based on tumor budding data; low (PBMC; *n* = 13, NILs/TILs; *n* = 11), intermediate (PBMC; *n* = 11, NILs/TILs; *n* = 7) and high (PBMC; *n* = 10, NILs/TILs; *n* = 9), and compared levels of various T cell subsets in circulation, NILs and TILs between them. We did not find any significant differences between CD4^+^ T cell subsets in circulation, NILs or TILs (Figures 7D–F). Moreover, there were no significant differences in the levels of other CD4^+^ T cell subsets, including TIM-3-expressing CD4^+^ T cells, between patients with different tumor budding status (data not shown).

## Discussion

Studies have reported correlations between increased T cell infiltration of various tumors and improved responses to therapies and favorable disease outcomes (29). Interferon-γ-secreting cytotoxic T cells, T-helper 1 (Th1) cells, natural killer cells and macrophages polarized to an M1 phenotype and DC1 dendritic cells are largely associated with favorable anti-tumor immune responses (30–32). While, Th2 cells, M2 macrophages, DC2 dendritic cells, myeloid derived suppressor cells (MDSC) and IL-10 and TGFβ releasing FoxP3^+^ Tregs are associated with immunosuppression (33, 34).

In CRC patients, high infiltration of CD3^+^ T cells, Th1 cells and cytotoxic T cells in the tumor center and invasive margins correlated with improved overall survivals and disease-free survivals, while lower T cell density was associated with poor prognosis (35, 36). In addition, high CD8^+^ tumor-infiltrating T cells were shown to be a favorable prognostic factor for right-sided colon tumors (37) and increased levels of CD4^+^ and CD8^+^ T cells in colorectal TME were shown to correlate with improved response to chemo-radiotherapy (38). Moreover, the presence of effector memory T cells within CRC tumors, defined by the presence of CD3, CD8, CD45RO, CCR7, CD28, and CD27 expression, was associated with absence of signs of early metastatic invasion (39). Therefore, evidence of an active immune response in the CRC TME was shown to be associated with prolonged survival (39).

We found that CD4^+^ T cells were significantly higher in colorectal tumors, compared with normal colon tissues. CD4^+^ T cells in circulation comprise mainly of naïve T cells, while in tissues comprise mainly of memory T cells. Galon et al. proposed that the immune landscape of CRC tumors can be considered as a robust predictor of patient survival and it may be used for histopahlogical classification of CRC tumors; they found that patients with high immune cell densities within the tumor and at invasive margins did not show recurrence (40). Importantly, transcriptomic profiling of immune subsets found in CRC tumors confirmed that immune cell infiltrates can affect disease outcomes as patients with prolonged disease-free survivals had distinct expression of genes related to cytotoxic T cells, T helper molecules and chemokine-related genes than patients with adverse disease outcomes (35). Therefore, comprehensive investigations are required to ascertain the role of immune cells in the TME and their effects on clinical outcomes of CRC patients. We reported high levels of different ICs and Treg-related markers in CRC TME, which would suggest their potential roles in carcinogenesis. Mechanisms of expansion/proliferation or trafficking of these CD4^+^ T cell subsets into tumor sites warrants further investigations.

Pre-existing Tregs in the TME expand upon antigen-specific activation in the presence of TGF-β and IL-10, which are found at high levels within the TME (41, 42). We found that CD4^+^CD25^+^FoxP3^+^ Tregs accumulate in colorectal tumors at significantly higher levels, compared to periphery and adjacent colon normal tissues. Moreover, these Tregs expressed high levels of Helios, indicative of highly suppressive and stable Treg function (43). Studies have shown that FoxP3^+^Helios^+^ Tregs have enhanced immunosuppressive characteristics, compared with FoxP3^+^Helios^−^ Tregs (44–46).

Majority of studies have associated Tregs with poor clinical outcomes in different cancers including CRC (47, 48), however some studies have also associated these with better prognosis in CRC patients (49–51). Saito et al. proposed that these results could be attributed to different subsets of tumor-infiltrating FoxP3^+^ Tregs, which include FoxP3^high^ and FoxP3^low^ Tregs; the former representing stable FoxP3 expression, while FoxP3^low^ non-suppressive Tregs secrete inflammatory cytokines and henceforth may be associated with improved clinical outcomes in CRC (14). In addition, it has been shown that tumor-infiltrating FoxP3^high^CD45RA^−^ effector Tregs, which express PD-1, are highly activated, express high levels of CTLA-4 and are associated with hyper progressive disease in patients with advanced gastric cancer (52). Based on these findings, we investigated differences in Helios and other IC expression between FoxP3^low^ and FoxP3^high^ PBMC, NILs, and TILs. We found that FoxP3^high^ Tregs express Helios at significantly higher levels than FoxP3^low^ T cells in the TME and in periphery of CRC patients, strengthening that Helios is a vital marker for suppressive Tregs in CRC. Additionally, FoxP3^high^ Tregs also showed significantly higher IC expression in the TME, indicating their highly activated states.

ICs are expressed on activated T cells including both Teff cells and Tregs (10), they are also highly expressed on dysfunctional and exhausted T cells, characterized by defective effector function and proliferation (53, 54). IC expression on T cells represent early activation or exhaustion due to prolonged exposure to residing antigens, which ultimately attenuate their effector functionality (55). Studies have shown that multiple inhibitory receptors are associated with T cell exhaustion but the specific mechanisms that administer their transcriptional and epigenetic development or their phenotypic identification remain to be fully elucidated. Notably, recent studies have proposed the HMG-box transcription factor TOX as a critical regulator of T cell exhaustion, which may be used to identify exhausted T cells (56). Additionally, investigating expression of other key transcription factors associated with T cell exhaustion, such as NFAT, EOMES, T-bet, FOXO1, and FOXP1 (55) in different IC-expressing CD4^+^ T cells would also ascertain their exhaustive states.

CTLA-4 is constitutively expressed on Tregs and modulates their functionality (57). Additionally, Tregs in the TME upregulate other multiple ICs including PD-1, LAG-3, TIM-3, and TIGIT (58); FoxP3^+^ Tregs expressing TIM-3/LAG-3 and PD-1 were shown to be highly suppressive (25, 59). TIM-3 expression on Tregs has been previously reported to show higher ability to suppress Th17 cells than TIM-3^−^ Tregs, while both can effectively suppress Th1 proliferation (60). We found that in the CRC TME, TIM-3 is mainly expressed on FoxP3^+^Helios^+^ Tregs, hence more potent suppressors of Th17 responses. In addition, we showed that PD-1, TIM-3, CTLA-4, and LAG-3 were mainly expressed on CD4^+^FoxP3^+^ Tregs in the TME, further reinforcing the suppressive role of these cells in the TME. tSNE representation, which enables visualization of high-dimensional data on a single bivariate plot, also confirmed the accumulation of Tregs and elevated IC expression on CD4^+^ T cells in the TME. Moreover, high IC expression corresponded with Treg populations in TILs.

Tumor budding is associated with vascular invasion and prognosis in colorectal cancer (61, 62). This study cohort consisted of CRC displaying different tumor budding characteristics, ranging from low to high. However, we did not find any differences in the levels of CD4^+^ T cells in periphery or TME across all patients with varying tumor budding status; thereby suggesting CD4^+^ T cells are not associated with tumor budding in CRC.

Following the accomplishments of PD-1 and CTLA-4 blockade, TIM-3 and LAG-3 are currently being explored in various pre-clinical and clinical trials to promote effective anti-tumor immunity for clinical benefits (63). This study provides comprehensive and simultaneous comparisons of expression levels of different ICs on CD4^+^ T cells, including Tregs, in the TME and periphery of CRC patients. IC inhibitors significantly improved survival in patients with MSI-H metastatic CRC. However, a significant proportion of patients show minimal response and do not benefit from ICIs (64). Additionally, the percentage of CRC patients who exhibit MSI-H/dMMR is generally low, around 12–15% of all cases (65), like in this study cohort (Table 1). Equating immune profiles of these patients with those who do not show microsatellite instability to find differences would therefore require a much larger patient pool. Moreover, the long-term prognosis of patients with advanced stage CRC remains poor despite efforts to develop novel chemotherapeutic and targeted therapy regimens. Predictive biomarkers for successful IC inhibition with clinical benefits are a necessity in such instances. Expression of ICs and their ligands in the TME have been proposed as such predictive biomarkers (66), and better understanding of the immune components can therefore assist in identifying robust biomarkers for response to therapy and also assist in targeted-therapies, tailor made on individual patient basis.

## Data Availability Statement

The raw data supporting the conclusions of this manuscript will be made available by the authors, without undue reservation, to any qualified researcher.

## Ethics Statement

The studies involving human participants were reviewed and approved by Qatar Biomedical Research Institute, Doha, Qatar (Protocol no. 2018-018) and Hamad Medical Corporation, Doha, Qatar (Protocol no. MRC-02-18-012). The patients/participants provided their written informed consent to participate in this study.

## Author Contributions

ST performed experimental work, data analysis, and wrote the manuscript. KM assisted with data acquisition and analysis. KM, MA-D, and MK contributed to sample collection, acquisition of patients' clinical data, and revising the manuscript. MA assisted in designing the study, contributed to sample collection, and revised the manuscript. EE conceived the idea, designed the study, obtained fund, analyzed and interpreted data, and wrote and revised the manuscript. All authors were involved in the final approval of the manuscript.

### Conflict of Interest

The authors declare that the research was conducted in the absence of any commercial or financial relationships that could be construed as a potential conflict of interest.
